# Histology of the Nerves

**Published:** 1882-11

**Authors:** 


### ARTICLE II.

### *Histology of the Nerves; a Contribution of the Latest Researches.*—(Continued).

By attentive observation one sees in the centre of Schwann's sheath a band, cylindrical in form, and refracting light less powerful than the myelin (which has just exuded); this cylindrical band is seen to extend a certain distance beyond the cut end of Schwann's sheath, and at that point is so pale and transparent as to be distinguished with difficulty.

This is the *axis-cylinder*, first discovered and described by Remark.*

* Froriep's "Neue Notizen." No. 47, 1837.

Thus we see a myelic nerve-tube is composed of the sheath of Schwann, the myelin and the axis-cylinder.

On changing the field one reaches a portion of the nerve-tube which has undergone no change in the relation of its structure. The double contour, due to the intact myelin, is seen. Tracing the tube further one encounters interruptions in the myelin, at more or less regular distances, in the form of traverse partitions, called *annular constrictions.* At the centre of the space between two constrictions the nerve-tube presents a nucleus, lying within a niche in the myelin and surrounded by a layer of protoplasm. As Sigmund Mayer * has pointed out, this protoplasm is not only granular but pigmented in the frog. In mammals it is simply granular, and but one nucleus is found in each inter-annular segment.

A properly-prepared nerve-tube should show the following details: At the annular constriction the nerve-tube terminates by a slight enlargement. This is due to a larger collection of myelin at that point than is found throughout the shaft of the *interannular segment.* This collection of myelin is contained within a pocket, so as to speak, formed by Schwann's sheath, thereby giving the former a convexity which rests within the concavity of the latter.

Between two of these convex enlargements—forming the extremity of two interannular segments, respectively—placed face to face, a biconcave meniscus is formed which, at first, appears perfectly transparent.

Attentive examination should discover the axis-cylinder traversing this space. The axis is crossed by a stria at the centre of the meniscus, or biconcave space, appearing the more brilliant as the objective-lens is raised from the slide.

At the extreme border of each convex enlargement of the myelin the concave fold or pocket of Schwann's sheath is seen.

---

* "Die Peripherische Nervenzelle und das Sympathische Nervensystem;" Archiv. f. Psychiatrie, 1876, p. 361.

A delicate nerve segment, treated by silver nitrate, shows the endothelial layer of Henle's sheath. Between the polygonal markings one sees, within this mass of nerve-tubes, a series of small Latin crosses, colored black. The longitudinal black bar is the axis-cylinder stained by the silver nitrate, whilst the transverse line corresponds to the annular constriction.

Attentive examination demonstrates, especially if the myelin have been rendered transparent by the action of glycerine, the alternating brown and black transverse striæ of the axis-cylinder, called *Frommann's lines*, after him who first described* them. These striæ are less marked, till final disappearance, the further from the constriction one examines.

Another silver nitrate preparation shows: Within the continuity of the annular segment, separated from the annular constriction, an enlargement which seems to be soldered to the axis-cylinder. It appears like two cones united base to base, through the axis of which the axis-cylinder passes. The surface formed by the union of the two cones is flattened. This body is called the *biconical enlargement*, is colored black, and on either of which is a somewhat clearer line, beyond which are seen Frommann's lines. In the normal condition of the nerve tube, this biconical enlargement corresponds in position to the annular constriction. In some preparations it is displaced, the axis-cylinder having become retracted within the nerve-tube. The fact that Frommann's lines diminish in size and clearness from the constriction as a centre when the condition of the nerve-tube is normal, whilst in some preparations the biconical enlargement is the centre, leads to the conclusion given above as to the normal position of the biconical enlargement.

In this same silver nitrate preparation a black ring is seen at the annular constriction. The existence of this

---

* "Zur Silberfærbung der Axencylinder;" Virchow's Archiv,, 1864 b. xxxi, p. 151.

black ring indicates, since axis-cylinder and biconical enlargement are displaced: first, tnat it is the sheath of Schwann which is thus colored at that point; second, the soldering together of two separate surfaces of that sheath, since it is known that whenever cellular elements (endothelial, epithelial, connective-tissue cells) are cemented together, treatment by silver nitrate demonstrates such union by black lines; third, that Schwann's sheath is formed of distinct segments, soldered together at each annular constriction. Evidently, therefore, the biconical enlargement is not solidly united to Schwann's sheath, otherwise the displacement thereof with the axis-cylinder could not occur. Regarding the incomplete constrictions described by various histologists, I have never observed them in well-prepared specimens (in physiological extension, fixed by acid osmic, and disassociated with care), and believe them to be due to imperfect preparation.

If a nerve be hardened with acid osmic, and disassociation carelessly and with violence, fractures of the myelin often occur. At the point of fracture it is easy to recognize the axis-cylinder, denuded of myelin, in the centre of the tube, and to distinguish at the edges the double contour of Schwann's sheath.

In a nerve-tube which has been freshly removed from the body and properly prepared, one can see, if the sheath of Schwann remain intact, oblique niches or fissures in the myelin of each interannular segment, called the *incisures of Schmidt.** They are found at irregular intervals. The *cylindro conical* limbs of myelin which they separate cover one another like slate-tiles upon the roof of a house. These limbs terminate by very acute angles, on the one hand, at the internal surface of Schwann's sheath, and, on the other, at the surface of the axis-cylinder, over which they form, for a certain distance, a very slender sheath, as

* Schmidt, "On the Construction of the Dark or Double-Bordered Nerve Fibre;" in the *Monthly Microscopical Journal*, May, 1874, p. 200, first drew attention to these niches.

it were. The segments of myelin limited by the incisures are of variable length, and their extremities assume three forms: 1. One being conical, the other hollowed out as a conical cavity, for the reception of the cone of another segment. 2. Both ends conical. 3. Both ends conically concave. These are called, *cylindro-conical segments.* Sometimes incomplete incisures, *i. e.* those which do not extend to the axis-cylinder, are observed.

The relation of the interannular nucleus to these incisures, and the limbs which they separate, is not constant, being sometimes situated upon the cylindro-conical segment, at others, over an incisure.

In nerves disassociated in acid-osmic and properly prepared with picro-carmine, a certain number of axes-cylinder become disengaged from their myelinic and Schwann's sheath, and float away, perfectly isolated. They are rose-tinted, sometimes possess a deeper tint. Attentive examination would demonstrate that they possess a very delicate *untinted* border, whilst their median portion, colored red, presents an obliquely-decussating striation.

Properly-prepared transverse sections of a nerve show: 1. That many axes-cylinder have changed from the cylindrical to a stellate form, due to an alteration in the myelin (which has formed *boules*, and compress the axes-cylinder) produced by the acid osmic solution. 2. That some axes-cylinder preserve their normal circular form, colored red. Between the rose-tinted axis-cylinder and its myelinic sheath a clear, uncolored ring is seen surrounding the axis-cylinder. This uncolored ring corresponds to a sheath, first described by Maunther,* and named after him *Maunther's sheath.*

All the special histological characters of myelinic nerve-tubes which have just been described may be observed in a *living* nerve-tube, without the aid of any reagent. A

* "Beitræge zur Kennt. der Morpholog. Elemente des Nervensystems:" Academy of Science of Vienna, Vol. xxxix.

preparation of living frog's lung, according to Holmgren's method, demonstrates that a myelinic nerve-tube :

1. Possesses a *double contour.* This enunciation is just contrary to that of classical authors, who have claimed that the double contour was due to a coagulation of the myelin after section or death.

2. Possesses the same morphological divisions as have been described above.

*Morphological Considerations.*—We have seen that Schwann's sheath is not continuous, but forms segments which are soldered to each other.

We also know that the myelin is interrupted at the annular constrictions, where it is held by the pockets formed by Schwann's sheath.

But the axis-cylinder *is continuous* throughout the length of the nerve-tube; presents no interruption from one end to the other. The proof hereof is found in the fact:

1. That non-myelinic nerve-tubes exist which each possess an axis-cylinder, and in which, by the aid of such reagents as have already been employed for myelinic nerve-tubes, nothing comparable to annular constrictions can be found ; there are no distinct segments.

2. That myelinic nerve-tubes which have been deprived of their medulla (reduced to their Schwann's sheath and axis-cylinder) show no traces of segmentation.

3. That the treatment of myelinic nerve fibres with a solution of caustic potassa (40 : 60) demonstrates there is no soldering of the axis-cylinder.

Each interannular segment may morphologically be compared to an adipose cell, the Schwann's sheath corresponding to the cell membrane, the interannular nucleus to the cell nucleus (each being surrounded by a protoplasmic layer), and the myelin to the fatty matter of the cell.

The axis-cylinder is not a component of a single interannular segment, but is a *distinct entity* within the nerve-tube.

The protoplasmic layer is not limited to the vicinity of the interannular nucleus, but, throughout each segment, lines Schwann's sheath; at the extremity of the latter it is reflected upon the axis-cylinder, which it surrounds. At the point of reflection the protoplasmic layer of one segment is set back to back with that of the neighboring segment, whence is formed the *biconical enlargement.* I would fain believe that these two layers, which are set back to back, are united by a cementing substance similar to that found between certain cells devoid of membrane, for example, between the cardiac muscular cells.

The incisures of Schmidt arise from the development of the myelin in several cylindro-conical segments, whereby prolongations from the superficial to the deep protoplasmic layers are preserved instead of forming a single mass of myelin within each interannular segment.

*Blood Vessels and Lymphatics.*—Properly injected and prepared specimens demonstrate the existence of arteries and veins within tho *perifascicular* sheath, and of smaller arteries and veins and capillaries within the *intrafascicular* sheath. In the former the blood vessels form a network with long longitudinal meshes, which appears similar to that found in muscles, and possessing, like the latter, greatly elongated meshes, and their longitudinal primary branches are regularly intersected by transverse or oblique branches. The *laminated sheath* possesses no other vessels than those penetrating it on their way to the intrafascicular sheath. Within the latter, and beneath a network of larger blood vessels (such as just described for the perifascicular sheath, only smaller), a capillary network, with elongated meshes, may be seen. These capillaries terminate by loops. From the summit of the convexity of each loop shoots a capillary which, further on, forms another loop, and soon indefinitely. At other points the offshooting capillary takes its origin at the summit of the loop which has been formed by the vessel taking a return course and forming its loop in an inverse direction. Again, a branch may pursue its course a certain distance, anastomosing with any other vessel, and rejoin the capillary from which it took its origin. This arrangement in forked series is very characteristic, and the *tout ensemble* may be called the *chain-like* capillary system.

The structure of the capillaries of nerves is the same as of capillaries in general.

The arteries and veins of the intrafascicular sheath are separated from the nerve-tubes by the lamina of this sheath. The capillaries are only separated from the nerve-tubes by the intrafascicular tissue proper (adults). In young animals their walls are, here and there, in direct contact with Schwann's sheath.

*The Nutritive Plasma.*—Each nerve-tube is constantly bathed in a nutritive plasma, yielded by the blood vessels which surround it, and is contained in the interstitial space formed by the laminated sheath on the one hand, and, on the other, by the nerve-tubes (surrounded by their Schwann's sheath, blood vessels and connective-tissue.)

This nutritive plasma gains the axis-cylinder principally through the interannular constrictions, since the myelin does not allow the easy penetration of liquids.

The interannular constrictions are, then, the celloid channels through which nutritive materials are conveyed to the axis cylinder.

*Role of the Different Parts.*—Schwann's sheath evidently serves to protect and maintain the myelin in place.

The myelin plays the part of protector to the axis cylinder, preserving it from compression. In all probability it acts as an insulator to the latter.

The annular constrictions act the physiological *role* of preventing the displacements of the myelin, which otherwise would certainly occur when placed vertically.

As we have already seen, they are the channels through which nutrition is supplied to the axis-cylinder.

The incisures of Schmidt are undoubtedly concerned in preventing displacement of the myelin.

The axis-cylinder is the medium through which the nervous influence is conducted.

*Sympathetic Nerves, or Remak's Fibres.*—Remak* announced, in 1838, that he had found fibres in the sympathetic nervous system which should be considered as nerve-fibres devoid of myelin.

To day histologists, with scarcely any exceptions, are convinced of the existence of such fibres.

Remak's fibres are particularly found in great abundance in the nerves of the system of organic life, but they do not, however, exclusively or specially belong to that system.

They are found in all mixed nerves in numbers varying according to the species of animal and the nerves under observation.

They do not exist in the special nerves, for example, the optic. The olfactory nerve contains nerve-fibres devoid of myelin, but these latter are wholly special, and should be classified and given a special description under the head of the terminations of nerves in the organs of special sense.

It is only necessary to know where Remak's nerve-fibres are to be found: our description shall be made by those found in mixed nerves.

Nerves which contain many of these fibres possess a gray or gelantinous appearance. In a state of relaxation they do not present as marked a pearly aspect as other nerves. This modified appearance is due, in these as well as in the myelinic nerves, to zigzags which the fibres form in the interior of the nerves. Microscopic examination of a nerve taken from the spleen and disassociated in water shows that the fibres composing the nerve are not arranged like myelinic nerve-tubes, to wit: like javelins in a quiver, but ramify, anastomose, and form a network, the branches of which approach one another, the interspaces of which are unequal; the *resean* offers more resistance to teasing in one direction than in another. The meshes of the network are quite irregular, very narrow and elongated, and always have their long diameter parallel to the axis of the nerve.

---

* "Observations Anatomica et Microscopicæ de Sytematis Nervosi Structura ;', Berlin, 1838.

The interspaces are of very unequal thickness; sometimes they are extremely delicate, at others they have the diameter of a medium-sized myelinic nerve; they also present intermediary dimensions.

Upon these interspaces nuclei are to be seen. They are generally presented in profile, and then seen simply to be applied to the surface of the interspaces. Sometimes they are seen in the centre of the interspace and in full view. At other times, too, they seem to occupy the interior of the interspace in profile; this would seem to show they are placed in the interstice of the two united fibres constituting it. Their disposition is not regular, and the interval between them is variable.

If a nerve, largely containing Remak's fibres, be submitted to the action of acid osmic and disassociated, the first fact which attracts our attention is that Remak's fibres remain uncolored whilst the myelinic nerve-tubes, even of the smallest diameter, are colored black. We may, therefore, conclude that Remak's fibres contain no myelin, or, at least, not enough to be acted upon by the reagent. Acid picric specimens stained with picrocarmine, Remak's fibres show indistinct, irregular, granular, longitudinal striation of those fibres.

If a dog's pneumogastric be acted upon and disassociated in acid osmic, and then submitted to the action of picrocarmine, we first notice that Remak's fibres possess a well-marked longitudinal striation, of such a character that the fibres seem to be formed by a series of juxtaposed small filaments.

The nuclei (with their nucleoli) and their surrounding protoplasm are perfectly clear. The preparation demonstrates that the nuclei are always applied to the surface of the fibres; when they appear to be in their interior it is because a nucleus is situated at points where two Remak's fibres are about to be united or separated, and seems to be situated in the interior of this interspace, when really it belongs to one of the fibres and is applied to its surface.

Finally, this same specimen demonstrates that Remak's fibres are not connective tissue fibres, since the picro-carmine has feebly colored the former whilst the latter remains absolutely uncolored. If red aniline be used, all doubts are at once dissipated by the deep red color of the former and the absence of color in the latter.

Although I have made a great number of specimens, very recently, too, with silver nitrate, I have never seen any indication of the soldering of two cellular elements, or any transverse striæ in Remak's fibres. I, nevertheless, do not consider the question as definitely settled. Perhaps the organic salts of silver may give different results. I should add, I have used silver lactate with negative results.

In specimens, prepared after the ammonia bichromate method and stained by picro-carmine, we note that, aside from the characters above described, each large Remak's fibre is composed of several fibres of very small size, either soldered together or separated over a certain distance. In each elementary fibre a large number of round or oval vacuoles are to be seen; they are uncolored and characterized, all vacuoles, by less refringence than that of the medium in which they are formed in the interior, even, of fibres which have been enlarged at their site, whilst elsewhere they are delicate and have thus become moniliform.

Transverse sections of a dog's pneumogastric or sciatic nerve, after treatment with acid chromic and picro-carmine, show, within the laminated sheath and between the myelinic nerve-tubes, very small, red, granular, irregular islets, which correspond to the fibres of Remak. There is also one elongated islet, situated immediately below the nerve's sheath, and distinct from the rest; this represents the section of the sympathetic nerve, which, as we know, is intimately united to the vagus. In these red-stained islets we are able to distinguish, by the aid of high powers, a series of very fine circles, pressed closely together. These circles represent the transverse section of the fibrillæ constituting the fibres of Remak.

This confirms our observation of their fibrillary structure when we examined them lengthwise after having disassociated them in acid osmic.

These are all the positive facts which we have acquired upon the constitution of Remak's fibres. But there are certain problems to be solved and hypotheses to be formulated upon this subject.

The first problem is in relation to their fibrillary structure. It is probable, indeed almost certain, that the fibrillæ we have recognized correspond to the axis cylinder. But this notion does not suffice, and pushing our analysis further, we should ask whether the juxtaposed axes-cylinder are absolutely uncovered or surrounded by an envelope. I know no method which can give any exact results upon this subject; the elements in question are so delicate, it is difficult to pronounce definitely, consequently I must lay this question aside without any reply until I speak of the morphological constitution of Remak's fibres. Second question: Are the fibrillæ placed side by side, like arrows in a quiver, or are they united by some cementing substance? This question is confounded with another, in relation to the nuclei and protoplasm.

It has been impossible for us to distinguish above the nuclei any contour which corresponds to a membrane like that of Schwann. It might be claimed that such a membrane is so extremely delicate, possessed a refringence so close approaching the medium in which it is situated, as to absolutely escape detection. I shall nevertheless assert that, *a priori*, I do not beleive so. In fact, if it existed, this membrane would be the envelope of a cell, and if Remak's fibres were surrounded by cellular segments lined by membranes, analogous to the interannular segments of myelinic nerves, silver nitrate should disclose the soldering by black lines. But it does not. We are right in concluding that Remak's fibres do not possess any properly-called membrane.

This absence of all traces of soldering authorizes us to add that the nuclei do not belong to so many distinct cells, and that we have to do here with a cellular mass with several nuclei, analogous to cells with multiple nuclei found in other tissues.

The last problem is: What is the extent of the protoplasmic layers around the nuclei? Do they extend the whole length of the fibres like a coating of varnish, or, on the contrary, do they penetrate their interior? Either because we do not possess lenses of sufficient power, or a good method, it is impossible to arrive at a decisive result. Nevertheless, let me say, that, in the best preparations, the small circles corresponding to the transverse section of the fibrillæ, the latter seem to be embedded in a cementing substance.

My theory is that Remak's fibres are constituted by elongated protoplasmic masses, their nuclei crowded to the periphery, and the fibrillæ of the nerves are lodged in the midst of the protoplasm.

*Morphological and Physiological Considerations.*—The peculiar differences between myelinic and Remak nerve-fibres, which have been above described, are sufficient to disprove the long held opinion that the latter is nothing more or less than a myelinic nerve arrested in its development, remaining, indeed, in an embryonic state.

The fibres of Remak constitute a distinct system, especially designed for organic life. Nevertheless, they are not exclusively charged with the innervation of the viscera, since the nerves distributed thereto also possess a certain number of myelinic nerves. Probably the latter serve to connect the organs with the central nervous system, whilst Remak's fibres establish their relations with the sympathetic nervous system. I am not convinced, however, that all myelinic fibres originate from the cerebro-spinal axis, and I believe there may be nerve fibres which are absolutely organic, arising from the sympathetic system to be distributed to the viscera, which possesses myelin. I think these are found as follows: We very well understand that myelinic nerve-fibres, from the cerebro-spinal axis and found in nerves destined to the viscera, may so act upon the fibres of Remak, in the midst of which they are imbedded, that a certain number of the latter gradually acquire the character of myelinic nerve-fibres. Such an influence is incontestible ; I shall take occasion to cite examples *a propos* of the tissue of cicatrices. Furthermore, 'since the *invertebrata* possess only non-myelinic nerves, the nerves of the *vertebrata* are undoubtedly the result of a higher development; now, if a distinct organism of higher growth be provided with myelinic nerve-fibres, why should not an organic system overleap, in its turn, even this limit between the two orders of fibres, and, also, arrive at the point of possessing myelinic nerve-fibres?

Thus, it is plain, taking the standpoint from histology, we can no longer distinguish between nerves of organic and of animal life, since both orders contain, in varying proportions, myelinic and Remak's fibres. Indeed according to the last considerations we have just mentioned, it is even possible that myelinic nerve-fibres are purely organic.

We, therefore, readily comprehend that Bichat's classsfication is no longer admissible by histologists. We should, consequently, abstain from all physiological classification of nerves and nerve-fibres and substitute simply a morphological one, distinguishing the myelinic from the nerve-fibres of Remak.—*Buffalo Med. and Surg. Journal.*

